# Quantitative evaluation of simulated functional brain networks in graph theoretical analysis

**DOI:** 10.1016/j.neuroimage.2016.08.050

**Published:** 2017-02-01

**Authors:** Won Hee Lee, Ed Bullmore, Sophia Frangou

**Affiliations:** a Department of Psychiatry, Icahn School of Medicine at Mount Sinai, New York, NY 10029, USA; b Department of Psychiatry, Behavioural and Clinical Neurosciences Institute, University of Cambridge, Cambridge CB2 0SZ, United Kingdom; c Cambridgeshire & Peterborough National Health Service (NHS) Foundation Trust, Cambridge CB21 5EF, United Kingdom; d National Institute for Health Research Cambridge Biomedical Research Centre, Cambridge University Hospitals NHS Foundation Trust, Cambridge CB2 0QQ, United Kingdom; e Immunopsychiatry, Alternative Discovery & Development, GlaxoSmithKline, Stevenage SG1 2NY, United Kingdom

**Keywords:** Neural dynamics, Kuramoto model, Graph theory, Resting-state fMRI, Computational model, Criticality

## Abstract

There is increasing interest in the potential of whole-brain computational models to provide mechanistic insights into resting-state brain networks. It is therefore important to determine the degree to which computational models reproduce the topological features of empirical functional brain networks. We used empirical connectivity data derived from diffusion spectrum and resting-state functional magnetic resonance imaging data from healthy individuals. Empirical and simulated functional networks, constrained by structural connectivity, were defined based on 66 brain anatomical regions (nodes). Simulated functional data were generated using the Kuramoto model in which each anatomical region acts as a phase oscillator. Network topology was studied using graph theory in the empirical and simulated data. The difference (relative error) between graph theory measures derived from empirical and simulated data was then estimated. We found that simulated data can be used with confidence to model graph measures of global network organization at different dynamic states and highlight the sensitive dependence of the solutions obtained in simulated data on the specified connection densities. This study provides a method for the quantitative evaluation and external validation of graph theory metrics derived from simulated data that can be used to inform future study designs.

## Highlights

• We tested the convergence of simulated and empirical brain network topology.
• Simulated functional data were based on the Kuramoto model.
• Network topology was defined using graph theory metrics.
• Graph theory metrics of global network topology showed greater convergence.
• The solutions obtained in the simulated data depended on connection density.

## 1 Introduction

Graph theory has been widely used to assess the topological properties of structural and functional brain networks inferred from neuroimaging data (Bullmore and Sporns, 2009, Bullmore and Bassett, 2011, Rubinov and Sporns, 2010). A large number of studies have shown that functional brain networks exhibit economical small-world topology and a hierarchical modular organization (Ferrarini et al., 2009, Meunier et al., 2009, Salvador et al., 2005), which provide both efficient global information exchange at relatively low wiring costs between clustered nodes (Achard and Bullmore, 2007, van den Heuvel et al., 2008) and resilience to random error and targeted attack (Achard et al., 2006).

Recently, the use of whole-brain computational models (Deco and Kringelbach, 2014) has shown promise in enriching our understanding of the mechanisms contributing to the formation and dissolution of functional brain networks (Deco et al., 2011). Computational studies coupled with empirical neuroimaging data have demonstrated the role of multiple time-scales in the patterns of functional connectivity (Honey et al., 2007, Rubinov et al., 2009), the emergence of resting-state activity from the local dynamics through structural connections of a small-world organized network (Honey et al., 2007), and the structure–function relation of resting-state networks (Honey et al., 2009). They have also identified the role of local network oscillations (Cabral et al., 2011, Deco et al., 2009) and the contributions of coupling strength, signal propagation delay, and noise, to the activity and organization of resting-state networks (Deco et al., 2009, Ghosh et al., 2008a, Ghosh et al., 2008b). Whole-brain models have been applied to neurological and psychiatric disorders to examine the impact of disrupted structural connectivity on neural dynamics (Adhikari et al., 2015, Alstott et al., 2009, Honey and Sporns, 2008, van Hartevelt et al., 2014, Vasa et al., 2015) and on disease states (Cabral et al., 2013, Cabral et al., 2012a, Cabral et al., 2012b).

A fundamental property of the brain is the oscillatory nature of neuronal activity. Phase synchronization of this oscillatory activity links neurons into functional assemblies which may be distributed in space, but are ‘locked’ together by a common signal phase (e.g., Eckhorn et al. 1988; Singer and Gray, 1995). Synchronization and self-organization phenomena in the brain are thought to exhibit critical dynamics (Beggs and Plenz, 2003; Botcharova et al., 2014; Breakspear et al., 2010; Kitzbichler et al., 2009; Meisel et al., 2013, Meisel et al., 2012; Shew and Plenz, 2013; Shew et al., 2009). Critical systems are generally defined as systems that are close to the boundary between weakly synchronized states (dominated by noise that prevents information flow) and globally synchronized states (that are static and have no behavioral value). There are many different phase relations within and between functional neuronal assemblies that give rise to multiple potential functional configurations of the brain network. Oscillations in brain regions can show positive (in-phase) or negative (anti-phase) temporal correlations, which are just the simplest of the many possible phase states that can be adopted by the brain. Multistability refers to the property of the brain to switch among multiple available phase states in order to adapt to external or internal demands (Kelso, 2012). Metastability is another dynamical property of the brain that describes the simultaneous realization of the tendency of individual brain regions to function autonomously while constrained by their interactions with other regions with which they are functionally connected (Kelso, 2012). Studies using a range of theoretical and computational models have found evidence of metastability in the human brain arising from the interaction between structural and functional connectivity (Cabral et al., 2011, Cabral et al., 2014a, Deco et al., 2009, Hellyer et al., 2015, Hellyer et al., 2014).

For a broad application of computational modeling, especially in clinical populations, it is important to determine the degree to which whole-brain computational models reproduce the graph-based topological features of the empirical functional resting-state networks. Two approaches can be distinguished in modeling brain dynamics. One is based on the observation that long-range temporal correlations exhibit power-law decay in oscillatory amplitude modulation (Linkenkaer-Hansen et al., 2001). The other is based on the recognition that spontaneous field potentials occur in outbursts and propagate following the same rules as avalanches (Hahn et al., 2010; Petermann et al., 2009; Shew et al., 2009).

Here we focus on the former approach and model whole brain dynamics using the Kuramoto model (Kuramoto, 1984), which is considered the most representative model of coupled phase oscillators and is widely used in the neuroscience research (Cabral et al., 2012b, Cabral et al., 2014b, Hellyer et al., 2015, Hellyer et al., 2014, Schmidt et al., 2014, Vasa et al., 2015, Vuksanovic and Hovel, 2014, Yan and Li, 2013). We modeled resting-state functional connectivity (FC) using the Kuramoto model “wired” by the empirical structural connectome. The simulated FC matrix was tuned to best fit the empirical FC matrix. We applied graph theory to simulated FC and empirical FC data to characterize key topological features, and then we quantified and compared the difference, in terms of relative error, in graph theoretical measures between simulated and empirical networks.

## 2 Materials and methods

### 2.1 Empirical connectivity

We used empirical connectivity data derived from diffusion spectrum imaging datasets that were acquired from 5 healthy right-handed male participants (age 29.4±3.4 years) on an Achieva 3 T Philips scanner using a diffusion weighted single-shot EPI sequence (TR=4200 ms, TE=89 ms, 129 gradient directions max *b*-value 9000 s/mm^2^) (Hagmann et al., 2008). Resting-state functional magnetic resonance imaging (rs-fMRI) data from the same 5 participants were acquired in eyes-closed resting-sate on a Siemens Trio 3 T system using a gradient echo EPI sequence (TR=2000 ms, TE=30 ms) (Honey et al., 2009). A symmetric, weighted structural connectivity matrix of the 66 anatomical regions was derived by down-sampling the 998 regions of interest (ROIs) connectivity matrix published by Hagmann et al. (2008) (Supplementary Figure S1; Supplementary Table S1). We chose these structural connectivity matrices as they have been extensively used in a range of different computational models to discover emergent properties of resting-state functional connectivity (Cabral et al., 2011, Hellyer et al., 2014, Messe et al., 2014, Vasa et al., 2015).

An empirical FC matrix (Supplementary Figure S1D) was independently created for each volunteer by applying a number of preprocessing steps including averaging of the blood oxygen-level dependent (BOLD) signal across voxels in each of the 998 ROIs, linear trending of the signal for each ROI, and regressing of the global BOLD signal as described by Honey et al. (2009). The resting-state FC matrix was computed based on Pearson's correlations between the BOLD time series of all possible pairs of 998 cortical regions. Then, the individual correlation matrices across the datasets of the 5 volunteers were averaged to obtain the empirical FC data. The down-sampled FC between the 66 anatomical areas was obtained as the average of all interregional FC correlations at the ROI level (Cabral et al., 2011).

### 2.2 Simulated connectivity

Following the approach of Cabral et al. (2011), we produced simulated FC data based on the Kuramoto model constrained by structural connectivity (Acebron et al., 2005, Cabral et al., 2011, Kuramoto, 1984, Vasa et al., 2015). Each of the 66 nodes, representing one of the 66 anatomical regions described above, was considered as an oscillator, and connected to all other nodes in accordance with the empirical structural connectivity matrix derived as described above. The connection of each region to itself was set to zero in the structural connectivity matrix for the simulations. The phase at each node over time $\theta_{i}\left(t\right)$ is described by a set of coupled differential equation (Acebron et al., 2005, Kuramoto, 1984):

$$\frac{d\theta_{i}\left(t\right)}{\mathrm{dt}}=\omega_{i}+k\sum_{j=1}^{N}C_{\mathrm{ij}}\sin\left(\theta_{j}-\theta_{i}\right)+\eta_{i}\left(t\right)$$

where $\theta_{i}$ and $\omega_{i}$ denote the phase and intrinsic frequency of region i. $C_{\mathrm{ij}}$ is the relative coupling strength from node j to node i based on the empirical structural connectivity matrix, and k is the global coupling strength which scales all connections’ strength. The term $\eta_{i}\left(t\right)$ represents the noise.

Phases were initialized randomly. We set the intrinsic frequencies to be uniformly distributed with mean=60 Hz and standard deviation=1 Hz, which corresponds to oscillations within the gamma frequency range. Gaussian white noise with mean=0 and standard deviation=3 rad was added to the model for biological realism (Ghosh et al., 2008b). Simulations were run for 320 seconds (first 20 seconds were discarded to remove transient effects) with a time-step of 0.1 ms for a range of global coupling strengths (0.5≤*k*≤25 at a resolution of 0.5) using an Euler scheme. Time-delay couplings were not incorporated to reduce the computational load and model complexity so that the global behavior of the model could be controlled using one model parameter, the global coupling strength *k* (for comparison to the Kuramoto model with time delays, see Supplemental section 2). The sine of the phases generated by the Kuramoto model (Cabral et al., 2011) was transformed into hemodynamic fluctuations using the Balloon–Windkessel model (Friston et al., 2000). The simulated fMRI BOLD signal was then low-pass filtered at <0.25 Hz and down-sampled at 2 s to match the same temporal resolution of empirical functional data as in Honey et al. (2009). After global signal regression of the resulting BOLD time series, the simulated FC matrices were calculated for each of the global coupling strengths, *k*.

We assessed correspondence between the simulated FC and empirical FC data by calculating Pearson's correlations between their upper triangular parts (excluding the diagonal). We then identified the coupling strength that provided the best fit to empirical FC. In order to improve stability and reliability, we simulated 10 runs of fMRI BOLD time series (obtained from 320 s simulations, discarding 20 s initial transients) with varying random initial conditions. We assessed the extent to which structural connectivity overlaps with the best-fit simulated FC matrices and empirical FC matrix using the Jaccard index as a measure of similarity. The similarity analysis between the binary structural connectivity versus simulated matrices as well as versus empirical FC matrix is discussed in the Supplemental section 3.

### 2.3 Metrics of network dynamics in simulated data

We evaluated the global behavior of the model using the order parameter R(t) defined as

$$R\left(t\right)e^{i\phi (t)}=\frac{1}{N}\sum_{n=1}^{N}e^{i\theta_{n}(t)}$$

where *N* is the total number of regions within the network. The level of synchrony between phase time series is described by the amplitude of *R(t)* which varied from 0 (fully asynchronous state) to 1 (fully synchronized state). At the global level, network dynamics were characterized by the mean and the standard deviation of the amplitude of *R(t)* over time; we considered the mean *R(t)* and an index of global synchrony and the standard deviation of *R*(*t*) as an index of metastability (Cabral et al., 2011, Shanahan, 2010). We acknowledge that first-order Kuramoto models, as the one presented here, do not exhibit true metastability (Breakspear et al., 2010). The temporal variability close to the onset of synchrony in the present case arises from standard criticality (Breakspear et al., 2010). Despite this caveat, we use the term metastability further in the manuscript as this term is commonly used to refer to the standard deviation of *R*(*t*) in first order Kuramoto models (Cabral et al., 2011, Shanahan, 2010).

### 2.4 Distributions of phase-lock intervals

We assessed whether the distribution of phase-lock intervals (PLI) in simulated fMRI BOLD time series follows a power-law function (Kitzbichler et al., 2009). The distributions of PLI were calculated for all possible pairs of derivations of simulated fMRI time series for a range of global coupling strength for scale 1 (0.25–0.13 Hz) and scale 2 (0.13–0.06 Hz).

To compute a scale-dependent estimate of the phase differences between two times series, we followed the approach introduced previously (Kitzbichler et al., 2009) using Hilbert transform derived pairs of wavelet coefficients (Whitcher et al., 2005). The instantaneous complex phase vector for two signals $F_{i}$ and $F_{j}$ is defined as:

$$C_{i,j}\left(t\right)=\frac{W_{s}{\left(F_{i}\right)}^{†}W_{s}(F_{j})}{\left|W_{s}(F_{i})\right|\left|W_{s}(F_{j})\right|}$$

where $W_{s}$ denotes the *s*-th scale of a Hilbert wavelet transform and † its complex conjugate. A local mean phase difference in the frequency interval defined by the *s*-th wavelet scale is then given by

$${∆\phi }_{i,j}(t)=\mathrm{Arg}(\bar{C_{i,j}})$$

with

$$\bar{C_{i,j}}\left(t\right)=\frac{\langle W_{s}{\left(F_{i}\right)}^{†}W_{s}\left(F_{j}\right)\rangle }{\sqrt{\langle {\left|W_{s}(F_{i})\right|}^{2}\rangle \langle {\left|W_{s}(F_{j})\right|}^{2}\rangle }}$$

being a less noisy estimate of $C_{i,j}\left(t\right)$ where 〈∙〉 denotes the temporal average over a brief period of time $∆t=2^{s}8$ (Kitzbichler et al., 2009). Intervals of phase-locking can then be defined as periods when $\left|{∆\phi }_{i,j}\left(t\right)\right|$ is smaller than some arbitrary threshold, which we set to π/4 here. In addition, we require the modulus squared of the complex time average, $\sigma_{i,j}^{2}={\left|\bar{C_{i,j}}\right|}^{2}$, to be greater than 0.5, limiting the analysis to phase difference estimates above this level of significance (Botcharova et al., 2012, Kitzbichler and Bullmore, 2015, Kitzbichler et al., 2009, Meisel et al., 2013, Meisel et al., 2012).

To confirm the robustness of our approach, we also conducted parallel analyses using ordinary band-pass filtering in conjunction with the Hilbert transform for each frequency band corresponding to 0.25–0.13 Hz and 0.13–0.06 Hz (see Supplemental section 4).

### 2.5 Power-law fitting and goodness-of-fit test

We assessed the presence of power-law scaling in the PLI distributions for each scale. We estimated the power-law exponent α based on maximum likelihood estimation (Clauset et al., 2009), and then evaluated the goodness-of-fit of power-law distributed probability distributions based on the Kolmogorov–Smirnov statistics, which generates a *p*-value that quantifies the plausibility of the hypothesis that the distribution is power-law like. This goodness-of-fit test involves sampling the fitted distribution to generate synthetic power-law data sets (*n*=1000) with parameters derived from power-law fit, and then computing the Kolmogorov–Smirnov distance between each data and the fitted distribution, producing the distribution of Kolmogorov–Smirnov distances expected if the fitted distribution is the true distribution of the data. A *p*-value is calculated as the proportion of artificial data sets showing a poorer fit than fitting the observed data set. When the *p*-value is close to 1, the data set can be considered to be drawn from the fitted distribution; otherwise the hypothesis might be rejected (Clauset et al., 2009, Meisel et al., 2013, Touboul and Destexhe, 2010).

### 2.6 Graph theoretical analysis of empirical and simulated data

We applied graph theoretical measures to two simulated FC matrices: one being the optimally simulated FC and the other at maximal metastability. The simulated and empirical FC matrices were each thresholded into an undirected binary graph network at each connection density across the 1–100% range, at 1% increments. We avoided a single arbitrary threshold that results in a set of graphs with varying number of edges (van Wijk et al., 2010). Instead, we constructed binary graph matrices that contain the same number of edges at any given threshold for a reliable comparison of the topological characteristics between graphs (Achard and Bullmore, 2007, Bassett et al., 2008). In addition to examining graph metrics over the entire range of connection densities (1–100%), we chose 14 binary graphs in the range of 37–50% for further comparison. This choice was motivated by previous reports (Lynall et al., 2010) that connection densities in the range 37–50% were less sensitive to inter-individual variability. We calculated the minimum density at which all nodes became fully connected (none of graphs were fragmented), and we estimated the small-worldness of the individual graphs to identify the maximum density at which the minimum value of the small-worldness index was greater than 1 (Bassett et al., 2008, Lynall et al., 2010). The maximum density at which below 50% of the nodes was also considered since connections at higher costs are less likely to be biological (Kaiser and Hilgetag, 2006).

We computed global and local efficiency, characteristic path length, clustering coefficient, betweenness and eigenvector centrality, participation coefficient, small-worldness, and resilience, for each graph at each connection density. These metrics, representing topological network characteristics, were estimated using the Brain Connectivity Toolbox (Rubinov and Sporns, 2010). *Global efficiency* (*E_glob_*) is the inverse of the shortest path length between nodes. (Latora and Marchiori, 2001). The *Clustering coefficient* (CC) is a measure of network segregation and is defined by the fraction of the neighboring nodes that are connected to each other. The *Characteristic path length* is computed as the shortest average path length between all pairs of nodes. *Small-worldness* (SW) is defined as the ratio between normalized clustering coefficient and normalized characteristic path length. Normalization for CC and characteristic path length was achieved by averaging their corresponding values over 1000 randomized networks. In a small-world network, the CC is significantly higher than that of random networks while the characteristic path length is comparable to random networks (Bassett et al., 2008, Lynall et al., 2010). *Local efficiency* (*E_loc_*) is a nodal measure and is defined as the inverse of the average shortest path connecting the node of interest to all its all neighboring odes. *Betweenness centrality* (BC) quantifies the fraction of shortest paths traversing a node within the network. *Eigenvector centrality* (EC) is a measure of centrality whereby nodes have high eigenvector centrality if they connect to other nodes that have high eigenvector centrality. *Participation coefficient* (EC) is a measure of the diversity of a node's inter-modular connections, based on a six-module partition following Hagmann et al. (2008) (Supplementary Table S2).

To assess the *resilience* of each functional network, we simulated attacks on the network by removing nodes either in order of higher degree (targeted attack) or in random order (random failure) (Bassett et al., 2008, Lo et al., 2015, Lynall et al., 2010). We incrementally eliminated the nodes from the network from 0 to 100% in increments of 1%, and then recalculated the size of the largest connected component or global efficiency of the remaining network after elimination of each node. Robustness was estimated by the area under the curve of the size of the largest connected component or global efficiency versus the number of nodes removed (Achard et al., 2006, Lo et al., 2015). For curves describing change in the size of the largest cluster size, this value was normalized by *N(N*−1*)*/2 to consider the size of the network *N*, so that the maximum robustness is 1. More robust networks retain a larger connected component even when a large proportion of nodes have been removed (Cabral et al., 2012b).

### 2.7 Comparison of graph measures between empirical and simulated networks

Graph measures of simulated FC and empirical FC were compared using the statistical measure of relative error (RE) (Lee et al., 2012, Lee et al., 2006), defined as

$$\mathrm{RE}=\sqrt{\frac{\sum_{i=1}^{N}{\left(G_{i}^{s}-G_{i}^{e}\right)}^{2}}{\sum_{i=1}^{N}{\left(G_{i}^{e}\right)}^{2}}}$$

where *N* is the number of connection densities (i.e., *N*=100 for the 1–100% range or *N*=14 for the 37–50% range). $G_{i}^{s}$ and $G_{i}^{e}$ denote the resultant graph-measure values from the simulated and the empirical data, respectively. The RE assesses the relative change between the absolute error (the numerator in the equation of the RE) and the reference solution $G_{i}^{e}$ computed from the empirical FC. The RE is 0 when the results are identical, and a lower absolute value of the RE corresponds to a smaller difference in the graph measure between the simulated and empirical data. We calculated the RE values over the entire (1–100%) and selected (37–50%) range of connection densities. For the sake of completeness, we also computed the Pearson's correlation coefficient between the graph measures of the simulated FC and empirical FC (see Supplemental section 5).

## 3 Results

### 3.1 Network dynamics based on the Kuramoto model

**Fig. 1:**
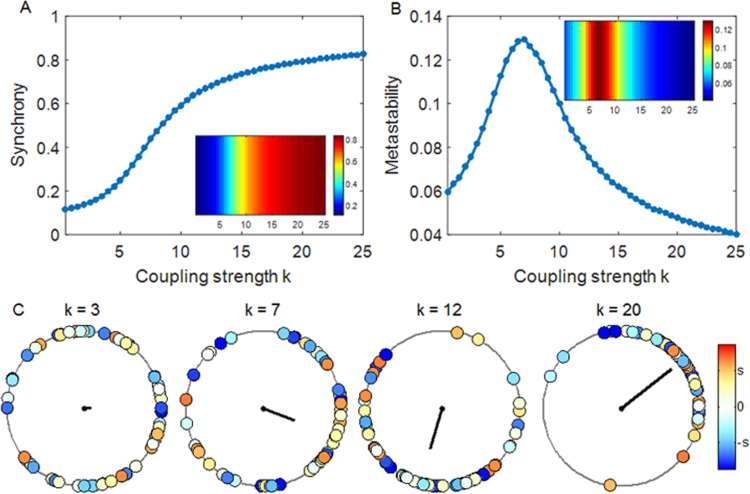
(A) Global synchrony (mean of the order parameter) and (B) Global metastability (standard deviation of the order parameter) of a representative Kuramoto model with varying global coupling strength *k*. Insets show the corresponding values as a blue-red colormap. (C) Color-coded phase circle diagram at *t*=300 s for coupling strength *k*=3, 7, 12, and 20. Solid line in black represents the amplitude of the order parameter, R(300 s)=0.1, 0.4, 0.5, and 0.8 for *k*=3, 7, 12, and 20, respectively. Angle and color in each oscillator represent phase *θ_i_* and frequency *ω_i_*−*ω_mean_*, respectively. As *k* increases, the oscillators corresponding to the 66 cortical areas transition from incoherence (left) to global synchrony (right). S denotes standard deviation of natural frequencies (*s*=1 Hz).

Fig. 1A and B show the dynamics of simulated brain activity as a function of the global coupling strength *k* derived from a representative Kuramoto model with oscillators (nodes) corresponding to 66 cortical areas described earlier. Fig. 1A shows the level of global synchronization measured by the order parameter *R*(*t*). For low coupling strengths, the phases of the oscillators (nodes) are desynchronized, resulting in a modular state characterized by small clusters of synchronized nodes. As the coupling strength increases, the network transitions from an asynchronous to an increasing synchronized state, as illustrated in Fig. 1C, with nearly all nodes being synchronized at higher coupling strengths. Fig. 1B shows the level of network metastability. Metastability is higher in intermediate states between asynchrony and synchrony, and is lower at higher coupling strengths when nodes become increasingly synchronized (Cabral et al., 2013, Cabral et al., 2011). Fig. 1C shows the behavior of the Kuramoto model with varying coupling strength at *t*=300 s. At low coupling strength (*k*=3), each node behaves incoherently resulting in a lower amplitude of the order parameter. However, as *k* increases, clusters of nodes begin to form resulting in an increase of the order parameter.

### 3.2 Identification of the optimal simulated connectivity matrix

**Fig. 2:**
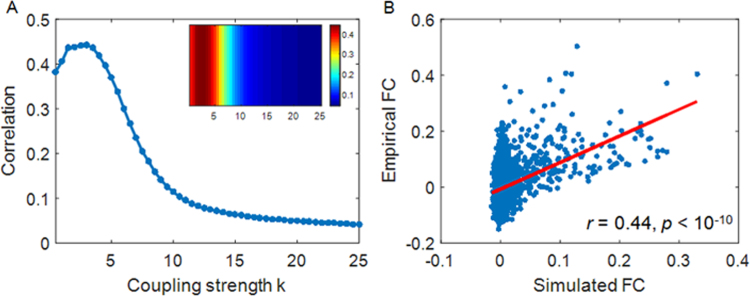
(A) Correlation between simulated FC (corresponding to the Kuramoto model shown in Fig. 1) and empirical FC as a function of the global coupling strength *k*. Inset shows the corresponding values by the blue-red colormap. (B) Scatter plot of empirical FC versus best-fit simulated FC at the optimum coupling strength (*k*=3). Solid line in red represents linear correlation between empirical FC and best-fit simulated FC (*r*=0.44, *p*<10^−^^10^).

We compared the simulated FC matrix and the empirical FC matrix by calculating the Pearson's correlations between corresponding elements of the upper triangular parts of the two matrices. Fig. 2A plots the correlation between the simulated FC (corresponding to the Kuramoto model shown in Fig. 1) and the empirical FC as a function of the global coupling strength *k*. The simulated FC matrix corresponding to the value of global coupling strength *k* at which the Pearson's correlation with empirical FC was maximal was identified as the optimal simulated FC matrix and corresponded to *k*=3. Fig. 2B shows a scatter plot between the empirical FC and the simulated FC matrix at the optimal coupling strength (*k*=3), giving a significant correlation between the simulated FC and the empirical FC (*r*=0.44, *p*<10^−10^). The averaged correlation fit between each of the ten best-fit simulated FC matrices and the empirical FC is 0.44±0.01 (mean±SD). These simulated FC data that were tuned with respect to empirical fMRI data were considered as the optimal model FC for further graph theoretical analysis.

### 3.3 Distributions of phase-lock intervals

**Fig. 3:**
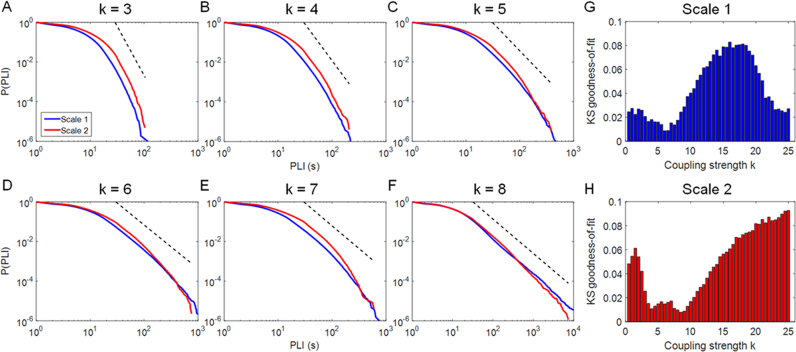
(A)–(F) The distributions of phase-lock intervals (PLI) from simulated fMRI time series (corresponding to the Kuramoto model shown in Fig. 1) for coupling strengths ranging from *k* 3–8. Dashed black lines indicate a power-law with exponent *α*=5.9, 4.7, 3.8, 3.2, 3.3, and 2.7 for (A)–(F), respectively, to guide the eye. (G) and (H) Goodness-of-fit of power-law distributions based on Kolmogorov–Smirnov (KS) statistics for scale 1 (0.25–0.13 Hz) and scale 2 (0.13–0.06 Hz). A KS value closer to 0 indicates better power-law fit.

As shown in Fig. 1B, a critical regime was observed for *k* values ranging from 5 to 10. However, the best-fit between simulated and empirical fMRI data was attained for *k*=3 (see Fig. 2A). Since this *k* value is below a critical range, we determined the distributions of PLI derived from simulated fMRI time series for scale 1 (0.25–0.13 Hz) and scale 2 (0.13–0.06 Hz) for a range of global coupling strength. We highlighted the PLI distributions for the *k* values spanning the range from near-critical (*k*=3) to critical point (*k*=8) in Fig. 3A–F. The corresponding KS goodness-of-fit values are shown in Fig. 3G and H. Although the distributions of PLI in a range of critical coupling strengths (*k* range=5–8) provided a better fit (lower KS values) overall, the differences in KS value for coupling strengths ranging from 3–8 were very small. For example, for scale 1, the KS value at *k*=3 is very similar to that of *k*=7. A high dissimilarity in KS values is seen in a “supercritical” range (*k*>15) and it seems attributable to the continuing presence of an apparent power-law distribution in this range.

Results obtained using ordinary band-pass filtering in conjunction with the Hilbert transform for each frequency band corresponding to 0.25–0.13 Hz and 0.13–0.06 Hz as done in wavelet-based PLI analysis are discussed in the Supplemental section 4.

### 3.4 Comparison of graph theoretical properties of empirical and simulated functional connectivity matrices

**Fig. 4:**
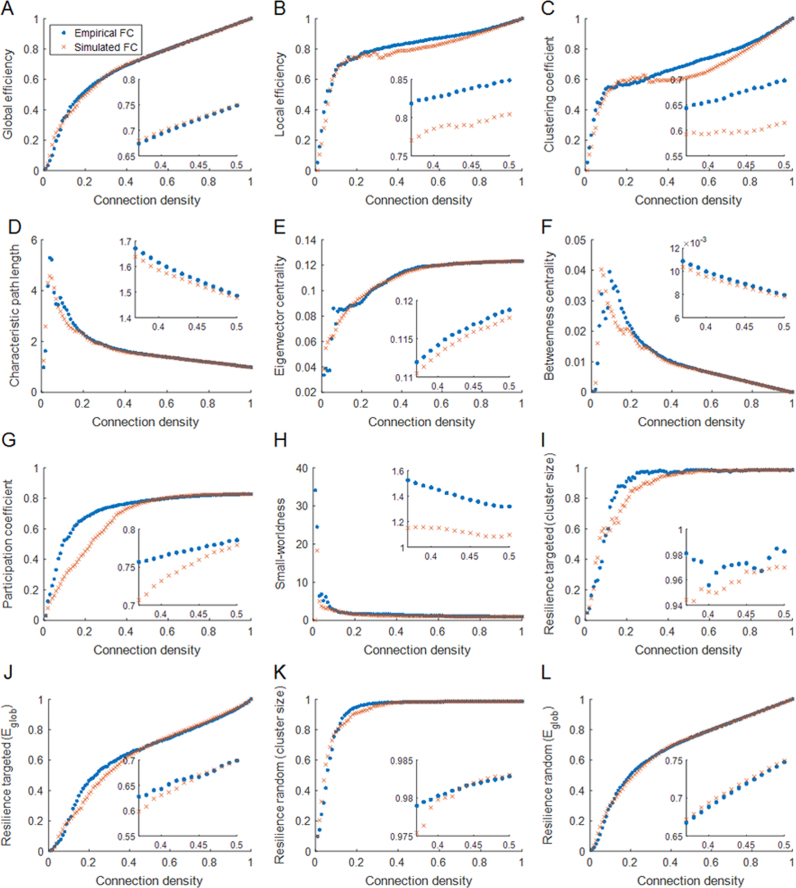
Comparison of the graph theoretical measures between the optimally simulated FC matrix (corresponding to Fig. 2B) and the empirical FC matrix as a function of the connection density. Insets show results for the selected range of connection densities (37–50%).

The topological features of optimally simulated and empirical functional networks were found to vary as a function of the connection density (Fig. 4). Overall, the topological properties of simulated functional networks become highly similar to those of empirical functional networks at higher connection densities. We found that global efficiency, betweenness centrality, and characteristic path length show a strong correspondence between the simulated and empirical FC (RE: 0.6±0.1% −4.6±1.1%), although the relative error for betweenness centrality and characteristic path length was greater than that of global efficiency over a range of 1–100% connection densities (RE: 13.3±3.4% −30.1±4.6%). Characteristic path length, betweenness centrality, and small-worldness decreased as connection density increased. Fig. 4 shows exemplary comparison results in the range of 37–50% connection densities. We also found that local efficiency also exhibits good similarity to between empirical and simulated data (RE: 6.1±2.2% −7.3±2.4%). In contrast, the relative error was higher for clustering coefficient (RE: 10.8±4.1% −16.9±6.7%), more so in the range of 37–50% connection densities than in the 1–100% range, unlike the other graph metrics. Eigenvector centrality and participation coefficient values of the simulated FC matrix, specifically over a range of 37–50% connection densities, are highly similar to those of empirical FC matrix (RE: 1.6±1.0% −2.2±1.6%).

Both the ten best-fit simulated FC matrices and the empirical FC matrix were tested for fragmentation to check whether this selected range of connection densities, in which the nodes are connected at least to one other node, is valid. We found that the fragmentation occurred at network density of 26%±5% in the simulated FC matrices (less than 37%), while the empirical FC matrix was fragmented at network density of 12%. We also observed that the small-worldness index was greater than 1 at network density of 50% in both the simulated and empirical functional networks.

### 3.5 Quantitative difference in graph theoretical measures between empirical and simulated FC

**Fig. 5:**
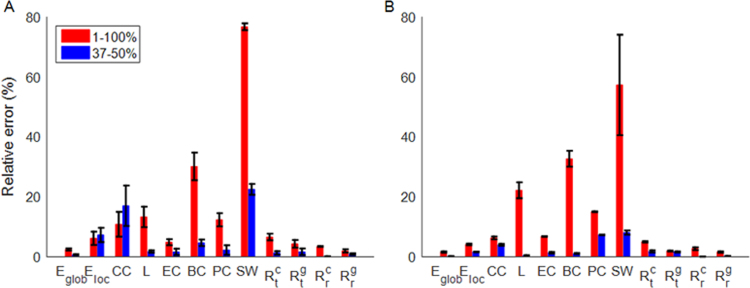
Relative error (RE) in percentage between graph theoretical measures of (A) best-fit simulated FC and (B) simulated FC at maximal metastability versus empirical FC for the entire (1–100%) and selected range of connection densities (37–50%). Bars and error bars correspond respectively to averages and standard deviations across the ten RE values. *E_glob_*: global efficiency, *E_loc_*: local efficiency, CC: clustering coefficient, *L*: characteristic path length, EC: eigenvector centrality, PC: participation coefficient, SW: small-worldness, $R_{t}^{c}$ and $R_{t}^{g}$ represent resilience to targeted attack in the size of largest connected component and global efficiency, respectively, $R_{r}^{c}$ and $R_{r}^{g}$ represent resilience to random failure in the size of largest connected component and global efficiency, respectively.

Fig. 5 shows the quantitative difference in graph theoretical measures between the empirical FC matrix and the simulated FC matrices at optimum coupling strength (Fig. 5A) as well as at maximal metastability (Fig. 5B) over the entire and selected range of connection densities. As shown in Fig. 5A, differences range from 2.4±0.4% to 76.7±1.1% over the entire range of connection densities, while the difference becomes smaller, ranging from 0.1±0.04% to 22.4±1.8%, over the range of 37–50% connection densities. Small-worldness yielded the largest difference across the entire range of connection densities. These higher RE values resulted primarily from a larger difference in normalized clustering coefficient (RE: 23.8±1.9% −62.2±0.9%) than in normalized characteristic path length (RE: 1.8±0.3% −8.2±1.2%) between simulated and empirical data. The smallest difference was observed in network resilience to random failure (global efficiency, $R_{r}^{g}$) in the 1–100% range and in network resilience to random failure (largest cluster size, $R_{r}^{c}$) in the 37–50% range. These results indicate that the simulated brain networks are approximately as resilient to random failure and targeted attack as the empirical networks. As shown in Fig. 5B, in comparison to the best-fit simulated FC, the simulated data at maximal metastability show reduced relative error for global and local efficiency, clustering coefficient, small-worldness, and resilience over a range of 1–100% connection densities. Increased relative error was however observed for characteristic path length, eigenvector centrality, betweenness centrality, and participation coefficient. For a range of 37–50% connection densities, the relative error decreased for all graph measures except for participation coefficient and resilience to targeted attack (largest cluster size). For further details about correlation between the simulated and empirical FC, see Supplemental section 5.

## 4 Discussion

This work provides a quantitative comparison of graph-theory based topological characteristics of simulated and empirical functional networks. For simulated data, we used a calibrated Kuramoto model based on 66 cortical regions constrained by white matter structural connectivity. We assessed the presence of critical synchronization using a power-law probability distribution of phase-lock intervals (PLI) derived from simulated fMRI data. We determined the extent to which graph theoretical properties of the simulated functional connectivity matrices arising from the Kuramoto model reproduce those derived from empirical resting-state fMRI data.

Kitzbichler et al. (2009) suggested that when the global coupling strength of simulated time series reaches criticality, they generate behaviors that most resemble those of empirical datasets. We found that power-law scaling of PLI distributions, an index of criticality, was present in the simulated fMRI data for *k* values ranging from 3 to 8; where *k*=3 is the optimal *k* value for the fit between empirical and simulated fMRI data and *k* range 5–8 is the critical range. We found that simulated data approached the critical regime at *k*=3 and best-fit with empirical data occurs in the critical border between desynchrony and synchrony (Fig. 3G and H). Power-law scaling was detectable for the range of *k*=3–8 while this was not the case for *k*>15. We infer that critical dynamics emerge in an anatomically realistic large scale network when the coupling strength is modestly positive in the range 3<*k*<8. This suggests that we can extrapolate the behavior of empirical data from simulated dynamics of an oscillating system at or close to a phase transition (Botcharova et al., 2012). Network dynamics in this paper were described in the Kuramoto model. Several new approaches have been proposed to simulate network dynamics that attempt to approximate neuronal ensemble behavior that may be more appropriate in the future. There are also alternative metrics to estimate power-law scaling in the simulated dynamics. For example, power-law scaling of so-called neuronal avalanches (spatially patterned bursts of synchronized firing across neuronal populations) has been demonstrated in neuronal slices, cultures, in vivo electrophysiological data in the macaque, and in human MEG and fMRI data (Alstott et al., 2009, Beggs and Plenz, 2003, Shew and Plenz, 2013, Shriki et al., 2013, Tagliazucchi et al., 2012). On the basis of these results on power-law scaling of PLI, we might expect to see a similar pattern of power-law scaling for neuronal avalanche size emerged in the Kuramoto connectomes over the same critical range of coupling strength, 3<*k*<8. This and other hypotheses about the links between connectome topology and simulated dynamics could be tested in future using the general methodological framework we have introduced here in the specific context of PLI scaling.

Global efficiency, characteristic path length and clustering coefficient were considered measures of global network organization. We found similar patterns for these measures when examining the simulated FC at best-fit and at maximal metastability. Global efficiency showed the most robust correspondence between empirical and simulated data across the entire range of connection densities. The same applied to characteristic path length particularly within connection density range of 37–50%, which may be less sensitive to inter-individual variation (Lynall et al., 2010). In comparison, the relative error for the clustering coefficient was larger across the entire range of connection densities.

With regards to measures that reflect local network properties, in the best-fit simulated FC, betweenness centrality, eigenvector centrality and participation coefficient showed robust correspondence between simulated and empirical data for connection densities ranging from 37–50%. Within this range, the relative error of these measures was smaller in simulated FC at maximal metastability with the exception of the participation coefficient. These results suggest that in simulated functional connectivity matrices, global efficiency can be confidently used to model the integration of brain networks. At the same time, caution is suggested with regards to the ability of simulated data to predict features of local information processing in brain networks as the relative error of these measures is more susceptible to changes in connection density. Nevertheless, the Kuramoto model would be suitable for studies on the characterization of hubs, which are nodes with high eigenvector centrality and participation coefficient, that play a crucial role in global brain communication (van den Heuvel et al., 2012, van den Heuvel and Sporns, 2011, van den Heuvel and Sporns, 2013). Several studies have reported disruption in hub organization in mental illnesses. For instance, in schizophrenia graph analyses of functional and structural networks suggest a less central role for frontal and parietal hubs (Lynall et al., 2010, Yu et al., 2011).

We assessed the resilience of the simulated and empirical functional networks to both random failure and targeted attack. We showed that the robustness of the simulated functional networks to random failure and targeted attack was highly comparable to that of empirical networks for both simulated matrices at best-fit and at maximal metastability. This indicates that the simulated brain networks are highly likely to behave like real brain networks in response to random failure and targeted attack. We also found that the relative error values in resilience to targeted attack were generally higher compared to random failure, suggesting that the simulated FC data can be favorably used to predict the effect on the global integrity of networks or the size of largest connected cluster in response to random elimination of nodes. These findings may be particularly relevant to the computational modeling of disorders such as schizophrenia where functional networks (matched for connection density) are topologically more random and more resilient to targeted attack than normal (Lo et al., 2015).

Small-worldness has been considered important in understanding brain functional networks, since it provides an architectural framework for both functionally specialized, topologically and anatomically segregated processes, and for functionally generalized, topologically and anatomically integrated processes (Achard et al., 2006, Latora and Marchiori, 2001, van den Heuvel et al., 2008, Watts and Strogatz, 1998). We found that small-worldness index had the highest relative error over a range of connection densities compared to the other graph measures for both at best-fit and at maximal metastability. In the case of best-fit simulated FC, a factor contributing to these higher relative error values was a larger difference in the normalized clustering coefficient (RE: 23.8±1.9% −62.2±0.9%) compared to the normalized characteristic path length (RE: 1.8±0.3% −8.2±1.2%). This is consistent with a relatively high difference in clustering coefficient as shown in Fig. 4C. This is in part explained by a large degree of variation in clustering coefficient observed in simulated functional networks as a function of the connection density. At lower connection costs, differences in normalized values for clustering coefficient and characteristic path length between simulated and empirical data contributed significantly to the relative error value in small-worldness. Our results indicate that simulated brain networks, at least those based on the Kuramoto model, are not likely to provide optimal solutions that simultaneously support locally specialized or modular processing and globally distributed or integrated processing (Bullmore and Sporns, 2009). Computational models could however be refined to reflect graph theoretical features of real brain networks, such as high levels of clustering and short path lengths, thereby yielding generative models with small-world architecture (Cabral et al., 2014a). Moreover newer developments in tract tracing have the potential to inform on the organizational principles of brain networks and thus to allow us to refine the most relevant range of connection densities for computational and graph theoretical modeling (e.g. Horvát et al., 2016).

In summary, we found that simulated data can be used with confidence to model graph measures of global network organization. We also highlight the dependence on connection density of the results obtained in simulated graphs, but not necessarily on the states of network. The relative error values in graph measures derived from the simulated FC at best-fit and at maximal metastability were comparable. This study demonstrates the value of computational models in assessing whole-brain network connectivity and provides a method for the quantitative evaluation and external validation of graph theory metrics derived from simulated data that can be used to inform future study design.

There are alternative approaches to that adopted here for comparing empirical and simulated fMRI resting-state networks. For example, Smith et al. (2011) simulated fMRI network data using the dynamic causal modeling (DCM) approaches to evaluate the performance of various connectivity metrics to recover the true underlying connectivity between nodes. Our approach was based on published resting-state fMRI network matrix inferred from Pearson's correlations (Honey et al., 2009). Although this is by far the most common method for generating resting-state fMRI network matrices, other metrics, such as partial correlation, regularized inverse covariance, or coherence, may also be useful in terms of testing the correspondence between simulated and empirical fMRI data and could be evaluated in future studies. The methodology presented in this paper provides a detailed framework for the evaluation of Pearson's correlation derived connectivity matrices and can be applied to any network data in future studies.
