# Supplementary material for: Quantitative evaluation of simulated functional brain networks in graph theoretical analysis
Source: Neuroimage. 2017 Feb 1;146:724–33. doi: 10.1016/j.neuroimage.2016.08.050 (PMC5312789; doi:10.1016/j.neuroimage.2016.08.050)
Supplement: Supplementary file 1 — Supplementary material [file mmc1.doc]

**Quantitative comparison of empirical and simulated functional brain networks using graph theoretical analysis**

**Supplementary Material**

**1. Empirical structural and functional connectivity**

Figure S1 shows empirical structural and functional connectivity datasets. The computational simulation was based on a network of the Kuramoto oscillators constrained by structural connectivity matrix describing the strength of white matter connections among 66 cortical regions (see, Table S1) defined using tractography of diffusion spectrum imaging, as illustrated in Figures S1A-C. Figure S1D shows empirical functional connectivity matrix.

**
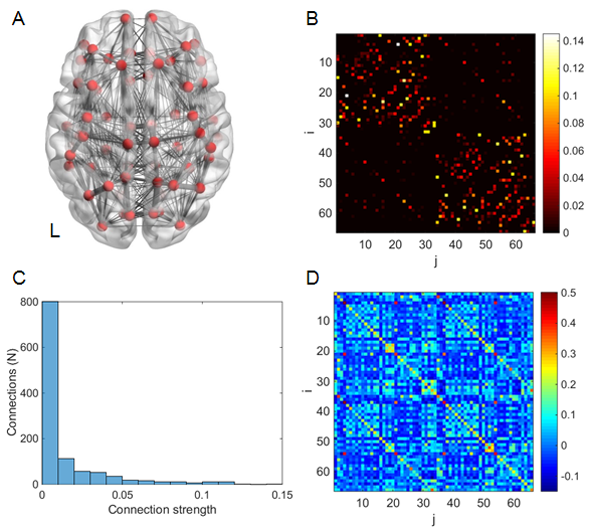
**

Figure S1. Empirical structural and functional connectivity data. (A) Structural network structure (superior view): the nodes and edges represent anatomical regions placed at their central coordinates and their mutual connections, respectively, (B) Connection strength matrix where i is the source region and j is the target (see Table S1 for 66 anatomical regions), (C) Histogram of the connection strengths, (D) Empirical functional connectivity matrix. L: left.

**2. The Kuramoto model with time delays**

We ran the Kuramoto simulations with a mean delay of 6 ms, assuming a mean conduction velocity of 10.7 m/s (mean tract length = 64.2 mm) which is within the range of physiologically realistic values estimated at 5-20 m/s (Waxman, 2006). The results in Figure S2A and B show the effect of time delays in global synchrony and metastability. The global behavior of the Kuramoto model with time delays changed due to the incorporation of time delays to the Kuramoto model. We next determined the simulated FC matrix that best fit empirical FC in order to compare the graph theoretical measures in terms of relative error and correlation coefficient. The results in Figure S2C and D indicate that the incorporation of time delays in the Kuramoto model does not seem to impact the relative error and correlation coefficient in graph theoretical measures.


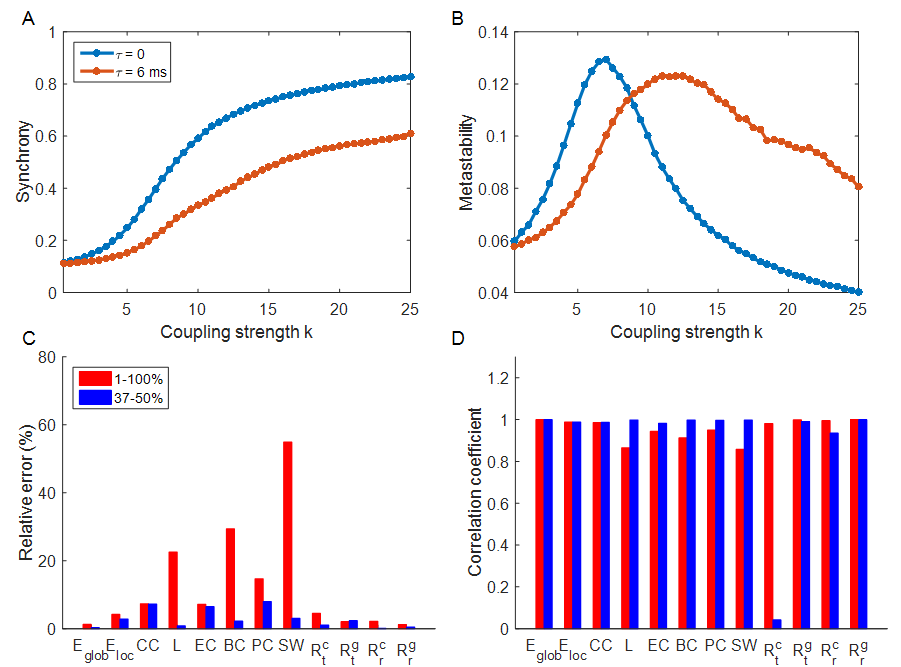


Figure S2. (A) Global synchrony and (B) Global metastability of a representative Kuramoto model with a mean delay of 6 ms for a range of global coupling strength. (C) Relative error (RE) in percentage and (D) Correlation coefficient (CC) between graph theoretical measures of empirical FC versus best-fit simulated FC matrix derived from the Kuramoto model incorporating time delays. E_glob_: global efficiency, E_loc_: local efficiency, CC: clustering coefficient, L: characteristic path length, EC: eigenvector centrality, PC: participation coefficient, SW: small-worldness, $R_{t}^{c}$ and $R_{t}^{g}$ represent resilience to targeted attack in the size of largest connected component and global efficiency, respectively, $R_{r}^{c}$ and $R_{r}^{g}$ represent resilience to random failure in the size of largest connected component and global efficiency, respectively.

**3. Similarity between structural and functional connectivity**

To assess the extent to which structural connectivity overlaps with the best-fit simulated and empirical FC matrices, the Jaccard index was used to assess the similarity between the binary structural connectivity and simulated or empirical FC matrices. Whilst the simulated or empirical FC matrix is practically a fully connected graph, the structural connectivity is highly sparse. For the structural connectivity, we introduced a threshold, equal to 0, above which the elements were set to 1 (0 otherwise). The Jaccard index was calculated as a ratio of the number of common elements (links) shared by the two matrices to the total number of elements in either one of them. The values of the Jaccard index range from 0 (no overlap) to 1 (perfect overlap).

Figure S3 shows the similarity between the binary structural connectivity and empirical or simulated FC matrices. The binary graphs of best-fit simulated and empirical FC matrices over the range of 1-100% were respectively compared to the binary graph of structural connectivity thresholded above 0, corresponding to a connection density of 27% (Figure S2A). At the same sparseness level (27%) as shown in Figure S3A-C, we found the Jaccard similarity index of 0.51 ± 0.03 for simulated FC matrices (Figure S3B) and 0.31 for empirical FC (Figure S3C). The comparison results between two binary networks exhibit a high similarity between the structural connectivity versus the simulated FC matrices than versus the empirical FC matrix. These results indicate that the DTI network base for the simulations influences the simulated FC matrix to a large extent.


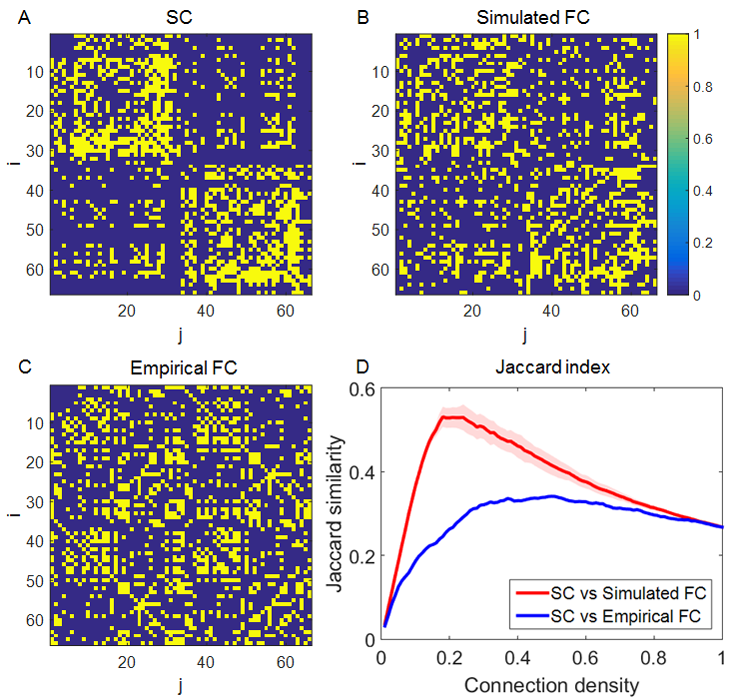


Figure S3. Binary graphs of simulated and empirical functional connectivity (FC) thresholded over the range of 1-100% were respectively compared to binary graph of structural connectivity (SC) thresholded above 0, corresponding to a connection density of 27% as shown in (A). Binary graphs of simulated and functional FC matrices at a connection density of 27% are also shown in (B) and (C), respectively. (D) Jaccard similarity between SC versus simulated FC matrices (n = 10) as well as versus empirical FC matrix as a function of the connection density. Red line and shaded error bars correspond respectively to averages and standard deviations across the ten Jaccard similarity index values between SC versus simulated FC matrices.

**4. Distributions of phase-lock intervals**

The transformation of functional neuroimaging data into phase representation has been previously performed using a wavelet analysis (Kitzbichler et al., 2009) and the computationally simpler Hilbert transform on band-pass filtered data (Glerean et al., 2012). We also analyzed the simulated fMRI time-series data using ordinary band-pass filtering and the Hilbert transform for each frequency band corresponding to 0.25 - 0.13 Hz and 0.13 - 0.06 Hz as done in the wavelet-based PLI analyses. Figure S4 shows that the band-pass filtering approach seems to yield comparable results. We note however that the wavelet-based analysis produces clearer power-law distributions in the high frequency range 0.13 – 0.25 Hz which corresponds to scale 1.


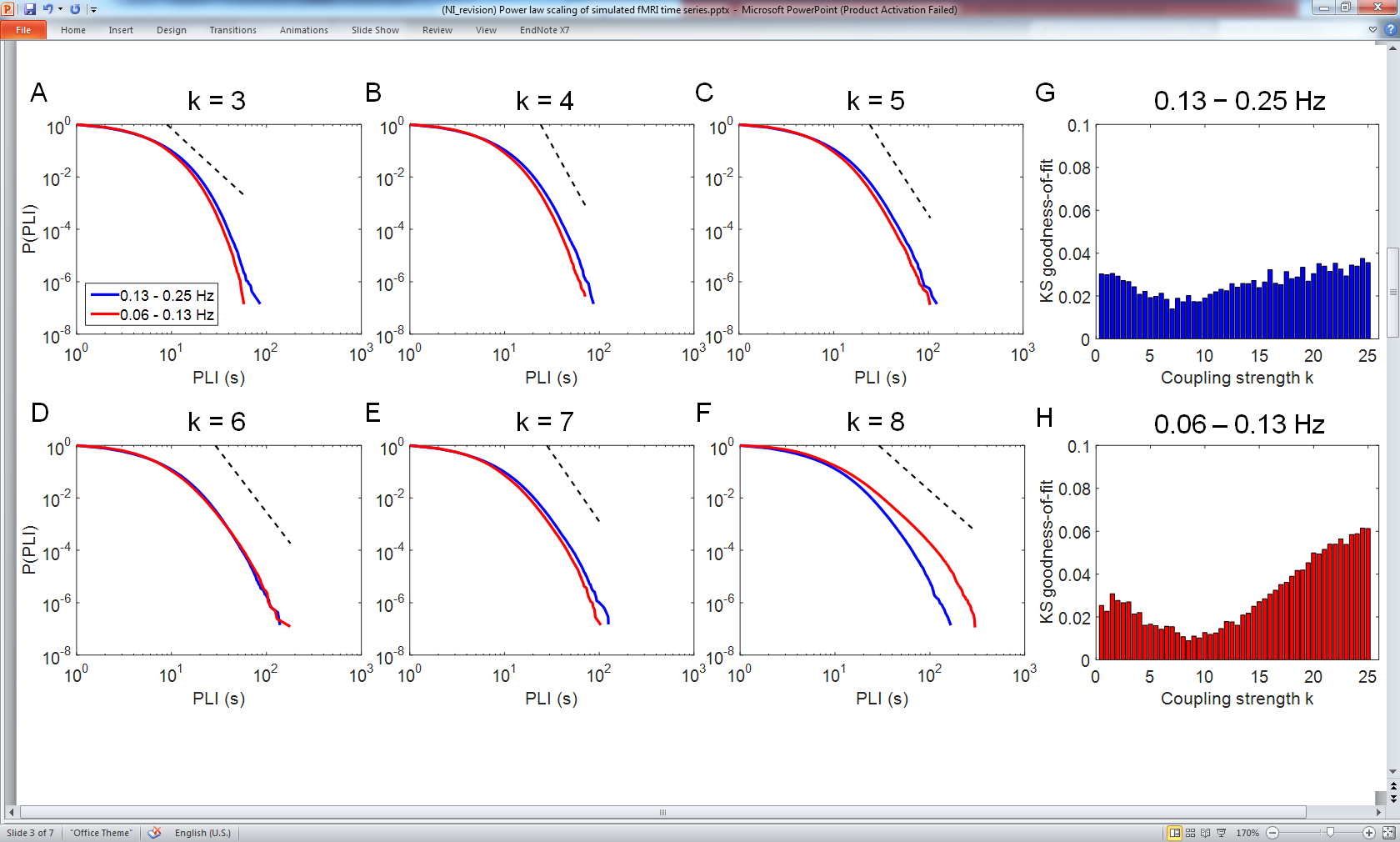


Figure S4. (A)-(F) The distributions of phase-lock intervals (PLIs) from simulated fMRI time series (corresponding to the Kuramoto model shown in Figure 1) using band-pass filtering and the Hilbert transform to acquire phase representation for coupling strengths ranging from optimal k (subcritical, k = 3) to critical value of k = 8. Dashed black line indicates a power-law with exponent α = 7.9, 7.5, 6.5, 5.7, 6.2, and 4.5 for (A)-(F), respectively, to guide the eye. (G)-(H) Goodness-of-fit of power-law distributions based on Kolmogorov-Smirnov (KS) statistics for the frequency band 0.13 − 0.25 Hz and 0.06 – 0.13 Hz. The KS value close to 0 indicates better power-law fit.

**5. Quantitative difference in graph theoretical measures between empirical and simulated FC**

In addition to relative error measure, we calculated correlation coefficient (CC) between the graph theoretical measures of best-fit simulated FC and empirical FC matrix. Our results in Figure S5A show that all graph measures exhibit strong correlation except for small-worldness in the 1-100% range (CC = 0.53) and resilience to targeted attack (largest cluster size) in the 37-50% range (CC = 0.28). The CC results between the simulated FC at maximal metastability and empirical FC matrix are shown in Figure S5B. We found strong CC values from the simulated data at maximal metastability highly similar to those of best-fit simulated FC data. At maximal metastability, the CC values decreased for characteristic path length in the 1-100% range and for resilience to targeted attack (largest cluster size) in the 37-50% range, and increased for small-worldness in the 1-100% range.


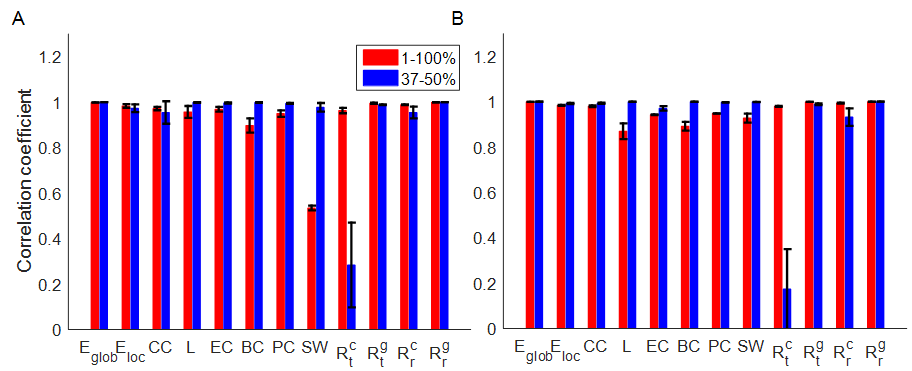


Figure S5. Correlation coefficient (CC) between graph theoretical measures of (A) best-fit simulated FC at optimum coupling strengths versus empirical FC and (B) simulated FC at maximal metastability versus empirical FC for the entire (1-100%) and selected range of connection densities (37-50%). Bars and error bars correspond respectively to averages and standard deviations across ten CC values. E_glob_: global efficiency, E_loc_: local efficiency, CC: clustering coefficient, L: characteristic path length, EC: eigenvector centrality, PC: participation coefficient, SW: small-worldness, $R_{t}^{c}$ and $R_{t}^{g}$ represent resilience to targeted attack in the size of largest connected component and global efficiency, respectively, $R_{r}^{c}$ and $R_{r}^{g}$ represent resilience to random failure in the size of largest connected component and global efficiency, respectively.

Table S1. Brain regions and abbreviations considered in the brain network model from Hagmann et al (2008). The numerical indices indicate the order of the respective brain regions in the right and left hemispheres.

| Brain region | Abbreviation | Right | Left |
| --- | --- | --- | --- |
| Bank of the superior temporal sulcus | BSTS | 1 | 34 |
| Caudal anterior cingulate cortex | CAC | 2 | 35 |
| Caudal middle frontal cortex | CMF | 3 | 36 |
| Cuneus | CUN | 4 | 37 |
| Entorhinial cortex | ENT | 5 | 38 |
| Frontal pole | FP | 6 | 39 |
| Fusiform gyrus | FUS | 7 | 40 |
| Inferior parietal cortex | IP | 8 | 41 |
| Inferior temporal cortex | IT | 9 | 42 |
| Isthmus of the cingulate cortex | ISTC | 10 | 43 |
| Lateral occipital cortex | LOCC | 11 | 44 |
| Lateral orbitofrontal cortex | LOF | 12 | 45 |
| Lingual gyrus | LING | 13 | 46 |
| Medial orbitofrontal cortex | MOF | 14 | 47 |
| Middle temporal cortex | MT | 15 | 48 |
| Paracentral lobule | PARC | 16 | 49 |
| Parahippocampal cortex | PARH | 17 | 50 |
| Pars opercularis | POPE | 18 | 51 |
| Pars orbitalis | PORB | 19 | 52 |
| Pars triangularis | PTRI | 20 | 53 |
| Pericalcarine cortex | PCAL | 21 | 54 |
| Postcentral gyrus | PSTC | 22 | 55 |
| Posterior cingulate cortex | PC | 23 | 56 |
| Precentral gyrus | PREC | 24 | 57 |
| Precuneus | PCUN | 25 | 58 |
| Rostral anterior cingulate cortex | RAC | 26 | 59 |
| Rostral middle frontal cortex | RMF | 27 | 60 |
| Superior frontal cortex | SF | 28 | 61 |
| Superior parietal cortex | SP | 29 | 62 |
| Superior temporal cortex | ST | 30 | 63 |
| Supramarginal gyrus | SMAR | 31 | 64 |
| Temporal pole | TP | 32 | 65 |
| Transverse temporal cortex | TT | 33 | 66 |

Table S2. Six network modules from Hagmann et al (2008).

| Module number | Member regions (LH) | Member regions (RH) |
| --- | --- | --- |
| 1 | lCUN, , lLING, lPARH, lPCAL, lPCUN | rCUN, rLING, rPCAL |
| 2 | lCAC, lISTC, lPARC ,lPC | rCAC, rISTC, rPARC, rPC, rPCUN |
| 3 | lBSTS, lENT, lFUS, lIP, lIT, lLOCC, lMT, lPSTC, lSP, lST, lSMAR, lTP, lTT |  |
| 4 |  | rBSTS, rENT, rFUS, rIP, rIT, rLOCC ,rMT ,rPARH, rPSTC, rSP, rST, rSMAR, rTP, rTT |
| 5 | lCMF, lFP, lLOF, lMOF, lPOPE, lPORB, lPTRI, lPREC, lRAC, lRMF, lSF |  |
| 6 |  | rCMF, rFP, rLOF, rMOF, rPOPE, rPORB, rPTRI, rPREC, rRAC, rRMF, rSF |

LH = Left hemisphere; RH = Right hemisphere; l = Left; r = Right
